# Designer pigs for xenogeneic heart transplantation and beyond

**DOI:** 10.1242/dmm.050177

**Published:** 2023-05-30

**Authors:** Eckhard Wolf, Bruno Reichart, Alessandra Moretti, Karl-Ludwig Laugwitz

**Affiliations:** ^1^ Gene Centre and Centre for Innovative Medical Models (CiMM), LMU Munich, 81377 Munich, Germany; ^2^ Walter Brendel Centre for Experimental Medicine, LMU Munich, 81377 Munich, Germany; ^3^ Medical Department I, Cardiology, Angiology, Pneumology, Klinikum rechts der Isar, Technical University of Munich, 81675 Munich, Germany; ^4^ DZHK (German Centre of Cardiovascular Research), Munich Heart Alliance, 80336 Munich, Germany

## Abstract

The 2-month-survival of a terminally ill patient who received a genetically modified pig heart has demonstrated that cardiac xenotransplantation could provide a therapeutic option for patients who cannot receive a human organ. Genetic engineering to overcome transplant rejection mechanisms, coagulation dysregulation and overgrowth of xeno-hearts has been the key to this success. The concept of exogenesis – the replacement of specific cellular populations and tissue structures of a pig heart with human cells – is a promising extension of xenotransplantation because it could further reduce immunological and physiological obstacles. Additionally, in the aim of preventing the need for heart transplant, tailored pig models mimicking monogenic cardiac disorders have been developed to test new cellular and molecular therapies. Thus, genetically engineered pigs provide a powerful platform for xenogeneic, exogenic and endogenic restoration of cardiac function.

## Teaser

**Summary:** Xenotransplantation of porcine hearts is a potential option for patients with terminal heart failure who cannot receive human organs. Genetic modifications of source pigs were essential for successful heart replacement experiments in baboons, paving the way to clinical studies.

## Introduction

Cardiovascular diseases (CVDs) remain the leading cause of mortality worldwide (Savarese et al., 2023). Current medical treatment options, such as β-blockers, angiotensin-renin-neprilysin inhibitors, aldosterone antagonists and sitagliptins, can slow down, but not revert, disease progression (Iacoviello et al., 2021). CVDs are therefore a major burden for health care systems; in the United States, adult cardiovascular spending increased from $212 billion in 1996 to $320 billion in 2016 (Birger et al., 2021).

For eligible patients with terminal cardiac failure, heart transplantation is the most effective treatment option. Unfortunately, the number of potentially life-saving donated organs does not match the clinical need (reviewed in Reichart et al., 2023). Under these circumstances, mechanical circulatory assist devices are the only available alternative, but at the cost of serious side effects, such as brain damage and infection (McNamara et al., 2021).

## Cardiac xenotransplantation as an alternative option

Cardiac xenotransplantation from genetically modified pigs has been discussed for decades as a potential alternative to allogeneic heart transplantation. Major advantages of pigs as organ sources include their similarity with humans in size and physiology, their high fecundity, and established techniques for their genetic modification and their maintenance under designated pathogen-free (DPF; see Glossary, Box 1) conditions. However, only recently have consistent long-term successes of porcine heart transplantations in baboons been achieved, with both heterotopic abdominal heart xenotransplantation (non-life-supporting; Box 1) (Mohiuddin et al., 2016) and life-supporting orthotopic heart xenotransplantation (Box 1) options being explored (Längin et al., 2018; Mohiuddin et al., 2022; Reichart et al., 2020). This paved the way to the first compassionate use of a heart from a cloned pig with ten genetic modifications in a terminally ill patient, who survived for 2 months post transplantation (Griffith et al., 2022). In view of the poor general condition of the patient before transplantation, intraoperative complications and the infection of the source pig with porcine cytomegalovirus (PCMV; Box 1), the 2-month survival of this patient is considered a success and shows that clinical xenogeneic heart transplantation is feasible.

“[…] the 2-month survival of this patient is considered a success and shows that clinical xenogeneic heart transplantation is feasible.”

**Box 1. Glossary**

**Activation of coagulation:** the process of blood clotting occurs in the course of hyperacute rejection of pig-to-primate xenotransplants and is triggered by endothelial cell activation and injury, resulting in haemostasis.

**Activation of complement:** this system of plasma proteins is triggered in primates by natural antibodies against specific carbohydrate antigens on pig cells and is a major driver of hyperacute rejection of pig-to-primate xenotransplants by promoting inflammation. In addition, complement activation is associated with ischaemia-reperfusion injury.

**CD40-CD154 (CD40L) co-stimulation blockade:** inhibits activation of T cells and is achieved by treatment with antibodies that block either CD40 on antigen-presenting cells or CD154 (CD40L) on T cells.

**Cold non-ischaemic perfusion:** is performed with an 8°C hyperoncotic cardioplegic solution containing erythrocytes, nutrition and hormones during and after explantation of the donor heart from the source pig to prevent cardiac xenograft dysfunction during the surgical procedure.

**Designated pathogen-free (DPF):** free of any known zoonotic pathogen to exclude the transmission of infections by xenotransplants.

**Heterotopic abdominal heart xenotransplantation:** an experimental model mainly used to evaluate the efficacy of immunosuppressive regimens and new combinations of genetic modifications. The porcine aorta is joined to the recipient abdominal aorta, and the porcine pulmonary artery to the recipient inferior vena cava in the abdomen. The transplanted heart is perfused via the coronary arteries and the coronary venous blood leaves the heart through the pulmonary artery trunk. The heart beats, but does not support the recipient's circulation. Recipient survival is maintained by their own heart.

**Insulin-like growth factor 1 receptor (*IGF1R*):** gene encoding the receptor for insulin-like growth factor 1 (IGF1), which modulates mitogenic and anti-apoptotic signals and is a key mediator of growth. Deletion of IGF1R in mouse embryos was shown to increase donor chimerism after complementation with mouse and rat pluripotent stem cells.

**Myogenic factor 5 (*MYF5*)/myogenic differentiation (*MYOD*)/myogenic factor 6 (*MYF6*):** genes encoding transcription factors belonging to the myogenic regulatory factor family of transcription factors. The ablation of these transcription factors in pig embryos prevents the formation of skeletal muscle. Using the myogenesis impaired embryos as hosts for complementation with human pluripotent stem cells can generate embryos with humanized muscle, as demonstrated up to day 27 of embryonic development.

**Orthotopic heart xenotransplantation:** replacement of the recipient's heart by the organ of a source pig.

**Porcine cytomegalovirus (PCMV):** a porcine roseolovirus (PRV), a common herpesvirus in pigs. In xenotransplantation, PCMV/PRV infection has been shown to significantly reduce the survival time of pig kidneys and hearts in preclinical trials with different non-human primates. Furthermore, PCMV/PRV has been transmitted in the first pig-to-human heart xenotransplantation and may have contributed to the death of the patient.

**Post-implantation growth control:** can be necessary to prevent detrimental intrinsic overgrowth of porcine hearts after orthotopic xenotransplantation. Overgrowth is inhibited by treatment with mechanistic target of rapamycin (mTOR) inhibitor.

**Protein C:** belongs to a key regulatory pathway of coagulation in the microvasculature. Protein C is activated by a complex of endothelial thrombomodulin (THBD) and thrombin in the circulation. This process is enhanced by the endothelial protein C receptor (EPCR). Activated protein C (APC) inhibits coagulation by inactivating factors Va and VIIIa. After xenogeneic organ transplantation, porcine THBD is a poor coactivator for human protein C. Therefore, transgenic source pigs expressing human THBD have been generated.

**Thrombotic microangiopathy:** involves the formation of platelet-rich fibrin thrombi in the microvasculature of xenogeneic organs due to molecular incompatibilities between thromboregulatory molecules present on pig endothelial cells and human/nonhuman primate clotting factors.

**Transient juvenile hypoglycaemia:** occurs in patients with growth hormone receptor deficiency (GHRD; Laron syndrome) and animal models for GHRD at juvenile age; glucose homeostasis normalizes after puberty.

## Genetic modification of source pigs

Multiple genetic modifications have been established in source pigs for xenotransplantation to overcome rejection mechanisms, inflammation, physiological incompatibilities and infection risks (Table 1). The question of how many genetic modifications are actually required to ensure long-term survival and function of porcine xeno-hearts remains a matter of debate (Kemter et al., 2020).

**Table 1.**
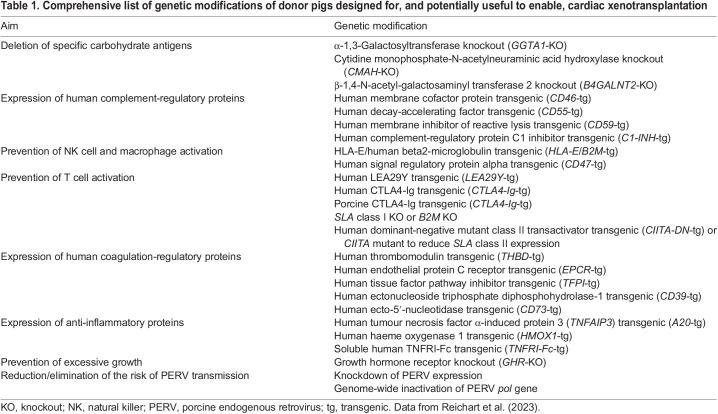
Comprehensive list of genetic modifications of donor pigs designed for, and potentially useful to enable, cardiac xenotransplantation

There is broad consensus that specific carbohydrate antigens on pig cells, such as galactose-α(1,3)-galactose (αGal), N-glycolylneuraminic acid (Neu5Gc) and a human Sd(a) blood group-like glycan, should be eliminated because they are recognized by preformed natural antibodies in humans and, for αGal and Sd(a), in non-human primates as well. Binding of these antibodies to their target leads to activation of complement (Box 1) and activation of coagulation (Box 1), leading to hyperacute rejection of the xenotransplant. This problem can be overcome by inactivating the genes *GGTA1* (encoding α-1,3-galactosyltransferase), *CMAH* (encoding cytidine monophosphate-N-acetylneuraminic acid hydroxylase) and *B4GALNT2/B4GALNT2L* (encoding β-1,4-N-acetyl-galactosaminyl transferase 2) (Table 1). Cells from these triple-knockout (TKO) pigs show very little human antibody binding (reviewed in Reichart et al., 2023).

An additive strategy is the expression of one or several human complement pathway regulatory proteins, such as CD46, in the pigs, which block different steps in the activation of the complement cascade. By combining this with specific pig antigen knockout strategies, hyperacute rejection of pig-to-primate xenografts can be completely avoided (reviewed in Reichart et al., 2023).

Another important issue is dysregulated coagulation in the xenotransplanted heart, eventually leading to thrombotic microangiopathy (Box 1) and graft failure. This can be avoided by transgenic expression of human coagulation regulating proteins (Table 1). So far, the most effective approach has been the expression of human thrombomodulin (THBD), which, in complex with human thrombin, activates protein C (Box 1), resulting in a strong anticoagulant function (Cowan and Robson, 2015).

**Fig. 1.**
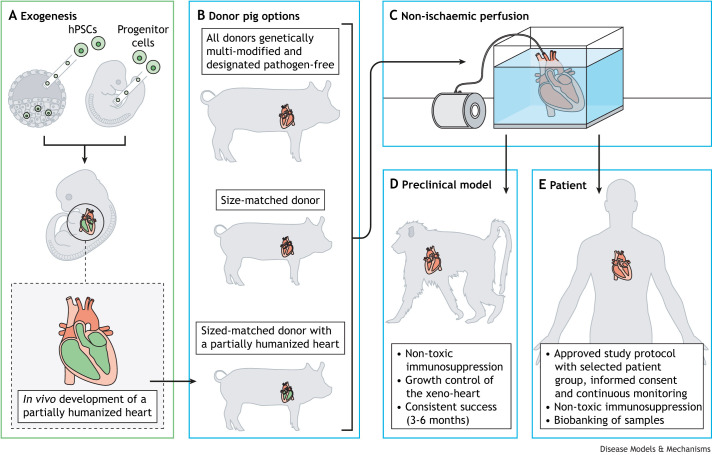
**Steps involved in the clinical translation of xenogeneic heart transplantation.** Modified from Reichart et al. (2023) under the terms of the CC-BY-NC 4.0 license. This image is not published under the terms of the CC-BY license of this article. For permission to reuse, please see Reichart et al. (2023). (A) Exogenesis is an additional step to generate partially humanized pig hearts by genetically engineering pig embryos to be deficient in the generation (or survival) of one or multiple cell lineages, then replacing this niche with human pluripotent stem cells (hPSCs) or progenitor cells at a later developmental stage *in utero.* (B) Donor pigs have been genetically multi-modified to reduce the risk of organ rejection, and precautions are taken to ensure the organs are designated pathogen-free and therefore not infected with zoonotic pathogens, such as porcine cytomegalovirus and porcine endogenous retrovirus. Additionally, to reduce the risk of organ overgrowth, studies have used size-matched donor pigs that have hearts more similar in size to human hearts. The final approach combines these considerations with exogenesis to generate a size-matched donor pig with a partially humanized heart. (C) Cold non-ischaemic perfusion is performed during and after explantation of the heart from the donor pig to prevent perioperative cardiac xenograft dysfunction during the surgical procedure. (D) Preclinical testing of xenogeneic heart transplantation in non-human primates has consistent success for 3-6 months when using therapeutics for immunosuppression and growth control of the xeno-heart. (E) Study protocols have been approved for future clinical studies with selective patient groups. Samples will be archived in biobanks to track molecular evidence of infection, including currently unknown pathogens.

Indeed, hearts from triple-modified pigs (*GGTA1* knockout and expressing human CD46 and THBD) survived consistently for up to 6 months after orthotopic transplantation into baboons (Längin et al., 2018; Reichart et al., 2020). In addition to genetic modification of the source pigs, cold non-ischaemic perfusion (Box 1) of the donor hearts, non-toxic immunosuppression of the recipients using CD40-CD154 (CD40L) co-stimulation blockade (Box 1), and post-implantation growth control (Box 1) of the xeno-heart using the mechanistic target of rapamycin (mTOR) inhibitor temsirolimus were key to the success in the baboons (Fig. 1) (Reichart et al., 2021). Additional transgenes, such as other complement pathway regulators (human CD55), human endothelial protein C receptor (EPCR; PROCR), human haeme oxygenase I (HMOX1) or human CD47 (Mohiuddin et al., 2022), did not provide a significant advantage over the combination of *GGTA1* knockout plus expression of human CD46 and THBD used by Längin et al. (2018). The only additional modification leading to an even longer survival in baboons was the knockout of the growth hormone receptor gene (*GHR*) to prevent excessive growth of the donor heart (Hinrichs et al., 2021; Mohiuddin et al., 2022). This modification was, therefore, also included in the source pig for the first compassionate use xenotransplantation in a patient (Griffith et al., 2022). However, *GHR* knockout pigs show several alterations, including transient juvenile hypoglycaemia (Box 1), marked obesity and changes in liver metabolism (Hinrichs et al., 2018; Riedel et al., 2020). Selection of a genetic background of source pigs that have hearts more similar in size to human hearts would therefore be preferable to the addition of *GHR* knockout. In appropriately sized donors, TKO of pig carbohydrate antigens plus expression of human CD46 and THBD may be the minimal set of genetic modifications required for clinical xenotransplantation trials (Reichart et al., 2023, 2021). Source pigs with much more complex modifications have been generated to counteract, for example, inflammatory reactions (Griffith et al., 2022; Yue et al., 2021), but these reactions can also be controlled by drug treatment. It is important to note that multiple genetic modifications may complicate breeding of the source pigs and there may even be unexpected interactions between transgenes or transgene products (Kemter et al., 2020).

“In appropriately sized donors, TKO of pig carbohydrate antigens plus expression of human CD46 and THBD may be the minimal set of genetic modifications required for clinical xenotransplantation trials.”

**Figure:**
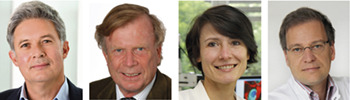
Eckhard Wolf, Bruno Reichart, Alessandra Moretti and Karl-Ludwig Laugwitz (left to right)

## Exogenesis – towards partially humanized pig hearts

A complementary technique to make xeno-hearts even more compatible would be the complete humanization of heart structures, such as the endothelium (Das et al., 2020). This approach, called exogenesis, is based on the formation of interspecies chimeras from host (pig) embryos that are genetically engineered to be deficient in the generation (or survival) of one or multiple cell lineages, thereby creating a niche(s) for donor cells of another species (human) to replace the missing cells (or even organs) (Fig. 1A) (Zheng et al., 2021). Genetic knockouts in pig embryos creating developmental niches for different tissues/organs are summarized in Table 2. Evolutionarily, pigs and humans are so distantly related that there is, however, a fundamental barrier to chimera formation of whole organs. Potential mechanisms representing this xenogeneic barrier include (1) apoptosis and cell competition, which can eliminate donor cells; (2) ligand-receptor incompatibilities between species; (3) differences in developmental timing; and (4) mismatches in cell adhesion molecules preventing the formation of adequate cell-cell junctions between human and host (pig) cells (Zheng et al., 2021). Overexpression of the anti-apoptotic factor BCL2 in human induced pluripotent stem cells (hiPSCs), which were engrafted into pig blastocysts lacking ETS variant transcription factor 2 (ETV2), a master regulator of haematoendothelial lineages, facilitated the generation of pig embryos [17 days post conception (E17)] with human endothelium (Das et al., 2020). Pig hearts with humanized endothelium would be an important achievement because the endothelium is the first target of rejection mechanisms after transplantation. Another possibility is to generate vasculogenesis-impaired porcine embryos via knockout of the kinase insert domain receptor (*KDR*) gene (Matsunari et al., 2020). Recently, porcine embryos with a human myogenic lineage were generated by complementation of pig blastocysts that were deficient in myogenic factor 5 (*MYF5*)/myogenic differentiation (*MYOD*; *MYOD1*)/myogenic factor 6 (*MYF6*) (Box 1) with hiPSCs lacking tumour protein P53 (TP53) (Maeng et al., 2021). *TP53* was inactivated to adapt the donor cells to the low *TP53* expression in the porcine host embryos, resulting in increased chimerism. Attempts to increase the inter-species chimera competency of human stem cells (naïve versus primed versus intermediate hiPSCs) have been comprehensively reviewed elsewhere (Zheng et al., 2021). Other attempts to improve interspecies chimerism target the host embryo, for example, by inactivating the insulin-like growth factor 1 receptor (*IGF1R*) gene (Box 1) (Nishimura et al., 2021). Systematic analyses of early porcine heart development should uncover new strategies for improving the efficiency of porcine blastocyst complementation with human stem cells, for example by humanizing essential ligands, receptors or adhesion molecules in the porcine host embryos.

“Systematic analyses of early porcine heart development should uncover new strategies for improving the efficiency of porcine blastocyst complementation with human stem cells, for example by humanizing essential ligands, receptors or adhesion molecules in the porcine host embryos.”

**Table 2.**
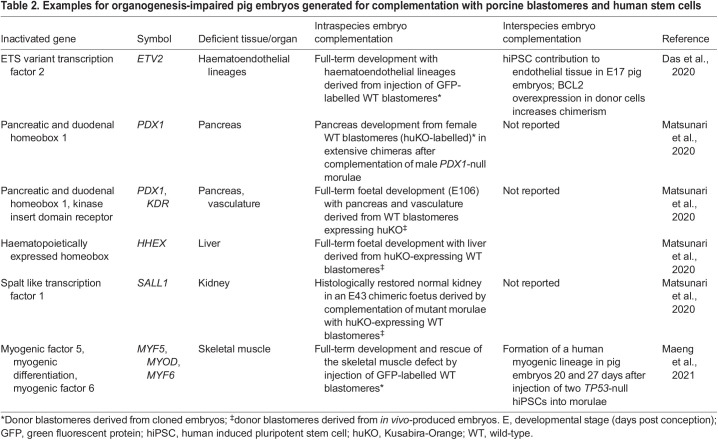
Examples for organogenesis-impaired pig embryos generated for complementation with porcine blastomeres and human stem cells

## Avoiding infection risks

An important issue for the clinical translation of xenogeneic heart transplantation is microbiological and, especially, virologic risk of infection (Fishman, 2022). Porcine endogenous retroviruses (PERVs) raised initial concern (Patience et al., 1997), but PERV infection has not been observed in patients treated with living pig tissues (Paradis et al., 1999) and PERV in source pigs can, in principle, be inactivated in a genome-wide manner using CRISPR/Cas9 (Niu et al., 2017). Furthermore, infection of the source pig with the porcine cytomegalovirus (PCMV) leads to early xenotransplant failure in baboons (Denner et al., 2020), and PCMV was detected in the first patient who received a porcine xeno-heart, which may have contributed to their early demise (Griffith et al., 2022). Stringent testing systems must be used to reliably exclude PCMV from source pigs (Halecker et al., 2022). Comprehensive lists of virus, bacteria, fungi and parasites that must be excluded from the source pigs have been published (Fishman, 2022). A recent Guidance from the Infectious Disease Community of Practice of the American Society of Transplantation (Mehta et al., 2023) summarizes the factors determining potential risk of zoonotic infections in xenograft recipients. The infection risks for xenotransplantation are considered manageable if sensitive and specific microbiological assays are used routinely for surveillance of source animals and xenograft recipients. Furthermore, appropriate samples from recipients, contacts and hospital staff must be archived for decades in order to track molecular evidence of infection, including currently unknown pathogens (Fishman, 2022).

“The infection risks for xenotransplantation are considered manageable if sensitive and specific microbiological assays are used routinely for surveillance of source animals and xenograft recipients.”

## Future perspectives

The ethical aspects of cardiac xenotransplantation have been discussed extensively, revealing no major objections (reviewed in Reichart et al., 2023). In general, intensive care unit-dependent patients with end-stage heart failure requiring continuous intravenous catecholamines are good candidates; secondary liver and kidney damage must be considered likely reversible and pulmonary hypertension medically treatable (Pierson et al., 2022). Initially, a xenotransplant may serve as a bridge to allotransplantation, and formal clinical cardiac xenotransplantation studies can be expected within the next few years. A prerequisite for these studies is safe source pigs raised in closed DPF units. There, the hearts will be explanted and sent in portable perfusion machines to the transplantation clinics.

The exogenesis approach is ethically more demanding due to the formation of animal-human chimeras. Different strategies have been developed to limit the differentiation potential of the donor hiPSCs to exclude the formation of brain and germ cells. Another approach would be complementation of the respective organogenic niche with human progenitor cells at a later developmental stage *in utero*. This ‘interspecies conceptus complementation’ would limit the risk of general chimerism, but requires developmental synchrony between the host organogenic niche and the injected donor progenitor cells, as well as application of the cells before the host immune system is formed (Wu et al., 2016). Porcine hearts with completely humanized endothelium could be a major advancement for patients with terminal heart disease. Although the exogenesis approach has the potential to generate organs with better compatibility, a disadvantage is that the whole procedure has to be done for each individual organ transplant, whereas complete xeno-hearts can be easily reproduced by breeding, once the right genotype for the source pigs has been established.

Finally, genetically modified pigs represent excellent models for translating innovative molecular and cellular therapies for acquired and inherited forms of cardiac diseases to reduce the eventual need for heart transplants (Box 2). Tailored porcine models with genetic mutations in structural heart muscle genes to mimic human hypertrophic or dilated cardiomyopathies can be used to test therapeutic efficacy of targeted approaches, such as CRISPR-based genomic engineering or cellular-based therapies. Overall, genetically tailored pigs have the potential to support xenogeneic, exogenic or endogenic restoration of a failing heart.

**Box 2. Targeting acquired and genetic heart disease with cell and gene therapies in genetic pig models**

With cardiovascular diseases and ischaemic heart disease in particular representing a huge burden for the health care system, inducing heart regeneration is one of the most critical unmet clinical needs. Myocardial infarction causes the hypoxic death of millions of cardiac muscle cells and extensive damage to the coronary vasculature, triggering a series of wound healing events including inflammation, myofibroblast activation and the formation of a permanent scar. This results in non-functional healing that ultimately leads to heart failure. Although certain drugs and mechanical devices can moderately improve cardiac function, they do not replace cardiac muscle cells and coronary vessels or abolish scar formation. Certainly, heart transplantation is the most durable and effective treatment option in regenerative medicine, providing complete physiological restoration. However, donated organs are scarce, and elderly patients are not eligible for organ transplantation due to their high comorbidities. This is why biotherapies have emerged as alternative strategies for heart repair (Desgres and Menasché, 2019). Cell therapies represent genetically engineerable solutions for tissue repair, given their capacity to sense and respond to extrinsic inputs. The grounds for human pluripotent stem cell (hPSC)-based cell replacement approaches to cure heart failure were laid by the discovery that cardiomyocytes and mesodermal heart progenitors can be efficiently differentiated from hPSCs *in vitro* and repair myocardium in mice and pigs (Foo et al., 2018; Romagnuolo et al., 2019; Poch et al., 2022). The preclinical studies in pig models demonstrated that cardiac progenitors have favourable results in halting heart failure progression (Poch et al., 2022).

Beside acquired forms of heart disease, tailored pig models of congenital heart disease, for instance with genetic mutations in structural heart muscle genes, are ideal for testing targeted therapies, such as gene therapy or exon skipping. In a pig model for Duchenne muscular dystrophy (Klymiuk et al., 2013; Stirm et al., 2021), restoration of the disrupted *DMD* reading frame by somatic gene editing resulted in expression of the formerly missing dystrophin and induced structural and functional improvements of skeletal muscle and myocardium (Moretti et al., 2020). Therefore, although xenotransplantation is an exciting prospect to treat end-stage heart failure, the pig heart can help develop alternative solutions to treat acquired or congenital heart disease before the extent of organ damage has progressed too far.
